# The Family Involvement in Care Questionnaire—An instrument measuring family involvement in inpatient care

**DOI:** 10.1371/journal.pone.0285562

**Published:** 2023-08-15

**Authors:** Anna Drakenberg, Kerstin Prignitz Sluys, Elisabeth Ericsson, Ann-Sofie Sundqvist

**Affiliations:** 1 Faculty of Medicine and Health, School of Health Sciences, Örebro University, Örebro, Sweden; 2 Department of Cardiothoracic and Vascular Surgery, Örebro University Hospital, Örebro, Sweden; 3 University Health Care Research Centre, Faculty of Medicine and Health, Örebro University, Örebro, Sweden; University of Verona, ITALY

## Abstract

**Background:**

Family involvement in care can be seen as a prerequisite for high-quality family-centered care. It has been identified to improve both patient safety and the quality of care by reducing patient complications and hospital length of stay.

**Objective:**

To develop and evaluate the content validity of a questionnaire measuring family involvement in inpatient care.

**Methods:**

The study followed a systematic approach in building a rigorous questionnaire: identification of domain, item generation, and assessment of content validity. The content validity index was calculated based on ratings of item relevance by an expert group consisting of seven senior nurses. Subsequently, 19 online cognitive interviews using the Think-aloud method were conducted with family members of former patients who had undergone open-heart surgery.

**Results:**

Five aspects of family involvement were identified, and the initial pool of items were selected from two preexisting questionnaires. The experts’ ratings resulted in item content validity of 0.71–1.00, and the scale content validity/averaging was 0.90, leading to rewording, exclusion, and addition of items. The pretesting of items through two rounds of cognitive interviews with family members resulted in the identification of three main problem areas: defining family involvement, misinterpretation of different terms, and underuse of the not relevant response option. The problems were adjusted in the final version of the questionnaire, which consists of 16 items with a four-point Likert scale and two open-ended items.

**Conclusions:**

The Family Involvement in Care Questionnaire has demonstrated potential in evaluating family involvement in inpatient care. Further psychometric properties regarding reliability and validity need to be established.

## Introduction

When a person needs hospital care, the persons family often has an important role in supporting the person’s emotional and physical well-being [1]. Involving family in care has several advantages, including improved patient safety [2] and quality of care [3, 4], and family members’ satisfaction with care [5]. Family involvement has the potential to reduce patient complications [6], hospital length of stay [7], and is a prerequisite for high-quality family-centered care [8]. Furthermore, it constitutes an important part of the framework for patient and family engagement in health and health care [9]. There are a variety of definitions of family involvement [1, 5, 10–12]. In previous research, patient involvement and family involvement have been closely linked [12] and, at times, used interchangeably [9, 13]. This project is grounded in the family-centered paradigm with a systemic approach to families [8]. In the family-centered paradigm, family involvement is not only a means of improving patient outcomes but also of supporting the whole family’s health [8, 14, 15]. Family involvement in care can be burdensome for the family, whereas support for the family is an important aspect of this concept [16]. “Family” is defined by Whall [17] as follows:

The family is a self-identified group of two or more individuals whose association is characterized by special terms, who may or may not be related by bloodlines or law but who function in such a way that they consider themselves to be a family (17, p. 241).

“Family involvement” is defined as taking part in the care of a family member and having one’s own needs of care met, whereas “taking part” refers to active involvement in care, decision-making and communication [12]. “Care” is specified in this study as the inpatient care provided during the patient’s hospitalization.

Elective open-heart surgery involves preoperative information and preparations [18] and postoperative intensive care and/or care in a step-down unit [19]. After the critical postoperative care period, the patient is referred to the ward prior to discharge, where rehabilitation at home begins [18]. The intensive care unit (ICU) stay may be prolonged if complications occur [20]. The need for surgery and postoperative intensive care puts a great deal of strain on both the patient and the family [21, 22].

When evaluating interventions promoting family involvement, previous research has focused on family involvement’s effects on patient outcomes [23]. In addition to evaluating patient outcomes, it is important to adequately measure if the intervention in fact has promoted family involvement from the family’s perspective. Therefore, valid measurements assessing family involvement in care should be used in research and clinical practice [23]. There are existing questionnaires measuring various aspects of the family experience of psychiatric care [24, 25], geriatric care [26–29], and intensive care [30]. In these context, the patient’s possibility to participate in their own care is reduced due to compulsory care, cognitive decline or critical illness and medical sedation. The family may therefore serve as a proxy for the patient. We believe that the family perspective on family involvement in these context differ from inpatient care settings where the patient is able to make decisions about their own care, treatment and can be active partners in their own care. To the best of our knowledge, there are no generic questionnaires measuring family involvement in the inpatient care setting. As described above, the standardized care pathway of open-heart surgical care results in that patients and their families are exposed to various care settings within the hospital. This care context enables, in our opinion, a suitable setting for testing the generic properties of a questionnaire applied in inpatient care. The purpose of the study was therefore to develop and evaluate the content validity of a generic questionnaire measuring family involvement in inpatient care.

### Materials and methods

#### Design

The design of this study was inspired by the guidelines for best practices for developing and validating measurement scales outlined by Boateng et al. [31], and reported according to the Consolidated criteria for reporting qualitative research (COREQ) checklist [32]. To create a rigorous scale, Boateng and colleagues [31] describe three phases: item development, scale development, and scale evaluation. These phases are further broken down into nine steps. The first three steps, concerning item and scale development, were used in this study:

- identification of domain and item generation,
- evaluation of content validity by an expert group rating relevance of items,
- pretesting the questions in cognitive Think-aloud interviews with the target population.

**Fig 1:**
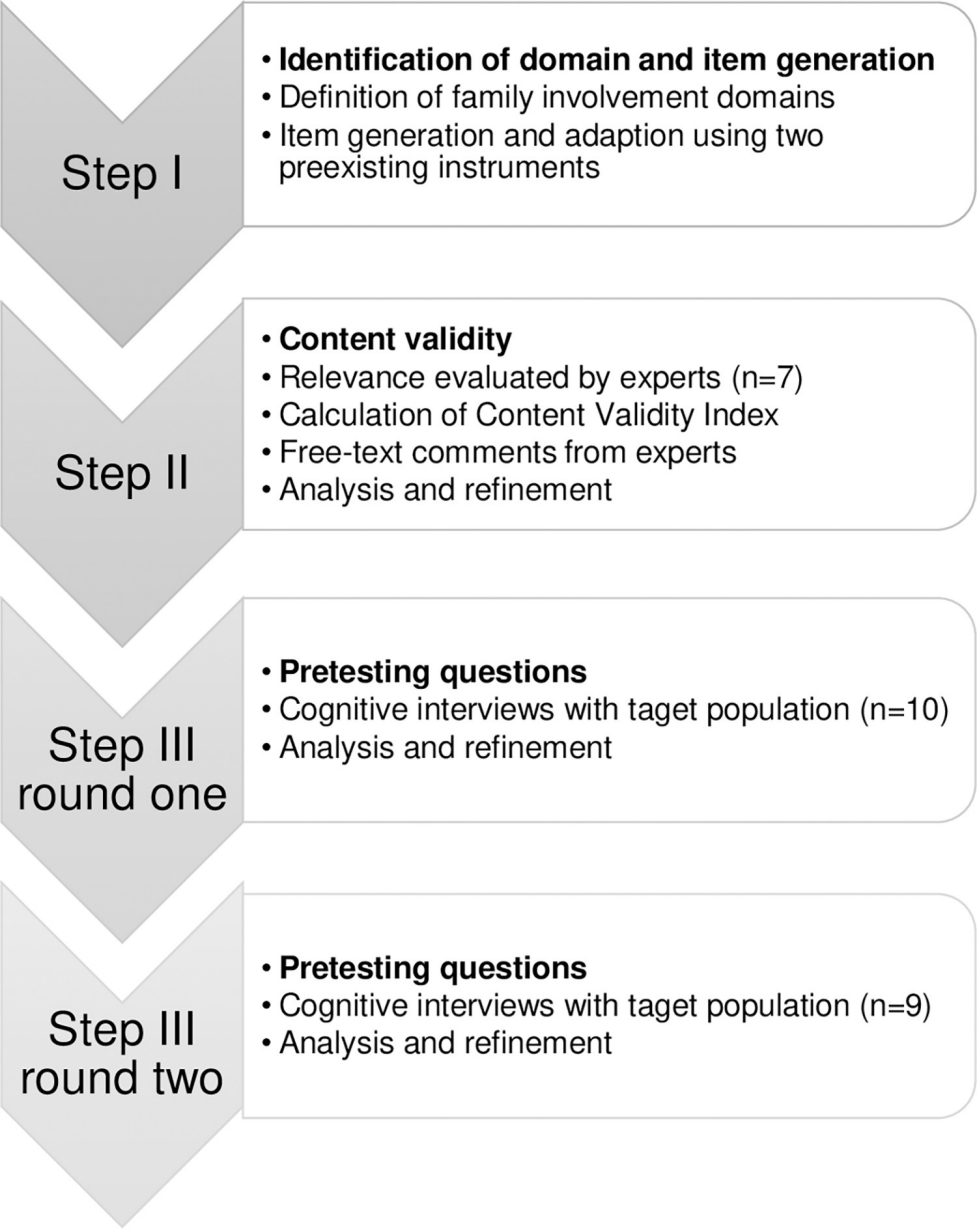
The scale development and testing process.

The scale development and testing process are displayed in Fig 1.

### Step I. Identification of domain and item generation

#### Identification of domain

The domain to be identified in this study was “family involvement” and the context “inpatient care”. The context was further specified as care related to open-heart surgery. The domain was identified in previous research using a broad scoping search strategy in the databases CINAHL and PubMed with the search terms family, involvement, participation, and engagement during the fall of 2019. The literature was deemed relevant if it concerned inpatient care and family involvement.

#### Item generation

Item development started by reviewing relevant literature regarding questionnaires and consulting leading researchers in the field of family nursing in Sweden who have developed questionnaires that evaluate patient and/or family involvement in care. The inclusion criteria regarding the questionnaires were that they had been developed in an inpatient care context relevant to surgical care and validated in Swedish.

### Step II. Content validity

#### Setting

Nurses in the expert group in step II were recruited from a cardiothoracic and vascular surgery department situated in a university hospital in Sweden. Approximately 500 open-heart surgeries are performed annually at the department, which consists of a surgical site, a thoracic ICU, and a ward. Patients come from the university hospital’s own catchment area and other parts of Sweden.

#### Evaluation by experts

The first version of the questionnaire was evaluated by seven senior nurses with extensive experience caring for patients and their families after open-heart surgery. This was done in December 2019 to determine item relevance in relation to family involvement in care. The nurses in the expert group were selected based on their years of experience as surgical nurses in the ward (i.e., a purposive sampling), and they were contacted by their work place e-mail. All nurses had the experience of being a family member to a person who had been admitted to inpatient care. The nurses holding a bachelor’s or master’s degree, as presented in Table 1, also had postgraduate education in surgical, vascular respectively operating room care.

**Table 1:** Demographics of the participants in the expert-group and in the cognitive interviews.

**Participants in expert group n = 7**		
VARIABLE		
Age in years, mean (SD)	41 (6.7)	
Experience as a nurse in years, mean (SD)	17.7 (8.1)	
Education		
Vocational degree (n)	3	
Bachelor’s degree (n)	2	
Master’s degree (n)	1	
Missing data regarding education (n)	1	
Experience of being a family member to a patient Yes /No (n)	7/0	
**Participants in cognitive interviews n = 19**		
VARIABLE	ROUND 1	ROUND 2
Total number	10	9
Sex: Male/Female (n)	3/7	4/5
Age in years, mean (SD)	61.9 (8.3)	64.3 (15.5)
Relationship with the former patient		
Partner* (n)	6	8
Child (n)	3	1
Relative (n)	1	—
Duration of interview in minutes, mean (SD)	24.7 (7.9)	20.0 (5.9)
Days between surgery and interview, mean (SD)	569.2 (101.2)	750.8 (115.3)

SD = Standard deviation, n = number

*Partner: spouse, domestic partner, partner

The content validity index (CVI) was used to summarize the degree to which the expert group agreed about item content validity. The assessment considered both the item (I-CVI) and scale (S-CVI/Averaging) levels [33]. The experts individually rated the relevance of each item in writing on a four-point Likert scale: (1) “not relevant,” (2) “somewhat relevant,” (3) “relevant” and (4) “highly relevant.” They were also able to add comments explaining their responses. The I-CVI score was calculated by counting the number of experts giving a rating of “3” or “4” on the four-point relevance scale and thereafter dividing that number by the total number of experts. The S-CVI/Averaging score was calculated by adding the sum of all I-CVI scores and thereafter dividing by the total number of items. A value of 0.78 for the I-CVI and 0.90 for the S-CVI/Averaging scores are considered excellent content validity [33].

### Step III. Pretesting questions

#### Cognitive interviews

To assess the appropriateness of the questions and response options for the target population, the questionnaire was evaluated in two rounds of cognitive interviews with family members. The cognitive interview method Think-aloud was used to analyze respondents’ cognitive processes while responding to the questionnaire [34]. In this method, well-designed questionnaires are formed through observation, reasoning, and cognitive interviews in which problems with the items are noted, and thereafter, refinements of the items are made. The sample size in cognitive interview studies should be small when conducted in several rounds, with no more than ten participants recommended in each round [34]. Data for step III were collected from January to June 2021. Due to the COVID-19 pandemic, cognitive interviews were conducted online using the videoconferencing platform Zoom Enterprise Video Communications (www.zoom.us, San Diego, CA).

#### Setting

The family members of former patients were recruited from the same setting as the nurses in the expert group, as described in Step II.

#### Recruitment and participants

The inclusion criteria for participants in the cognitive interviews were that they must be aged 18 years or older, a family member of someone who had undergone open-heart surgery, and be able to read, understand, and communicate in Swedish. To avoid the limitation of the participants’ range of experiences, no requirements regarding level of participation or time of presence at the hospital were made.

Recruitment started with a random selection of patients from the 2019 register of open-heart surgery at the university hospital. These patients received a letter with basic information about the study and were asked if a person in their family would be interested in participating. Former patients responded by providing contact information for presumptive participants. These family members of former patients who had undergone open-heart surgery were contacted with a letter providing full information about the study, including the objectives of the study and requesting their informed consent. A week later, they received a phone call to ask whether they had any questions and whether they were interested in participating in the study. Letters were sent out in several rounds to ensure that the number of twelve participants was not exceeded in any round. In total, thirty-six former patients were asked about presumptive participants, twenty-six of whom provided information about their family member. Out of these twenty-six family members of former patients, nineteen were willing to participate, four declined, and three could not be reached. No reason was requested for not participating. However, one of the four declining family members spontaneously stated that the reason they were not able to participate was that they had no e-mail address that could be used to send a link to a digital meeting and that they were not interested in having one. Ten of the nineteen participants were interviewed in the first round, and nine were interviewed in the second round (see Table 1).

#### Data collection

Think-aloud interviews were carried out online individually with the participants in their own homes or private offices. Each participant was interviewed at one occasion. Standardized instruction [35] and probes [34] were used. On two occasions, the participant had company at home, but this did not disrupt the interview. The purpose of the study was discussed and clarified before starting each interview. The interviews were conducted by the first author. Supervision and continuous feedback were provided by the second author, who had prior experience in using the Think-aloud method.

**Fig 2:**
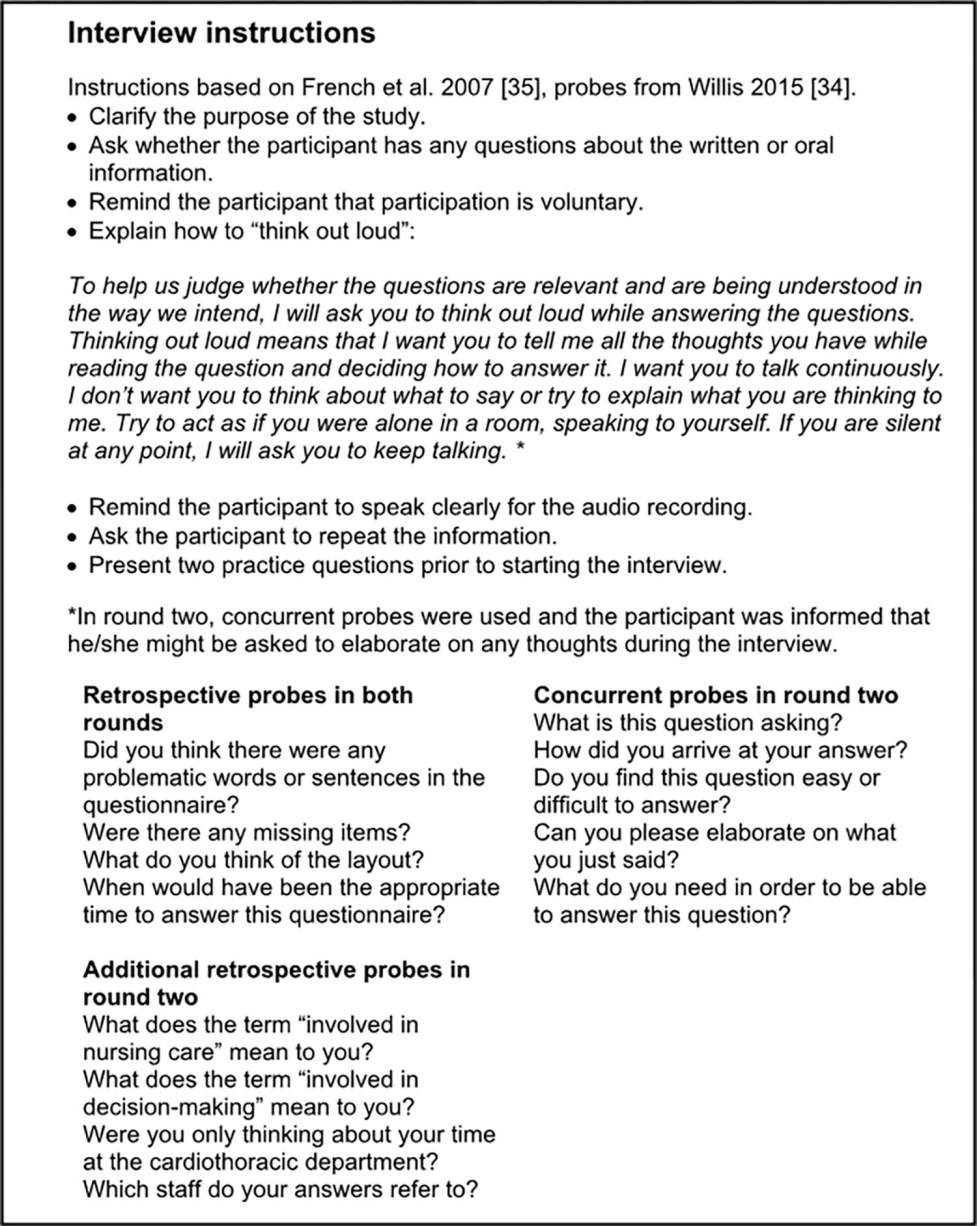
Interview instructions for cognitive interviews testing the Family Involvement in Care Questionnaire (FICQ).

The instructions for the interview process were pilot tested during the first interview, and no problems were identified. Problems with the probing strategy were, however, identified after the analysis of the first round. Therefore, the instructions were modified prior to the second round. In the first round of cognitive interviews, no concurrent probes besides the prompt “think out loud” were intended to be used. However, concurrent probes were used on twenty-six occasions, as pauses might indicate technical problems. This led to a change in strategy for the second round. In the second round, it was decided to include five concurrent and four additional retrospective probes in the instructions as a strategy to standardize the concurrent probes. The instructions are presented in Fig 2.

All interviews were audio-recorded. The participants were able to choose whether they wanted their video on or not during the interview, and thirteen out of the nineteen chose to have their video on. The participants could always see the interviewer. The questions were displayed using screen sharing, one at a time. The participants read the question aloud, verbalized their thoughts about the question/answer alternatives, chose an answer, and then indicated that they were ready for the next question. After the Think-aloud interview, the participant was asked to comment on any problematic wording or missing items and to give feedback on the layout and when they would have wished to receive the questionnaire.

#### Data analysis

**Fig 3:**
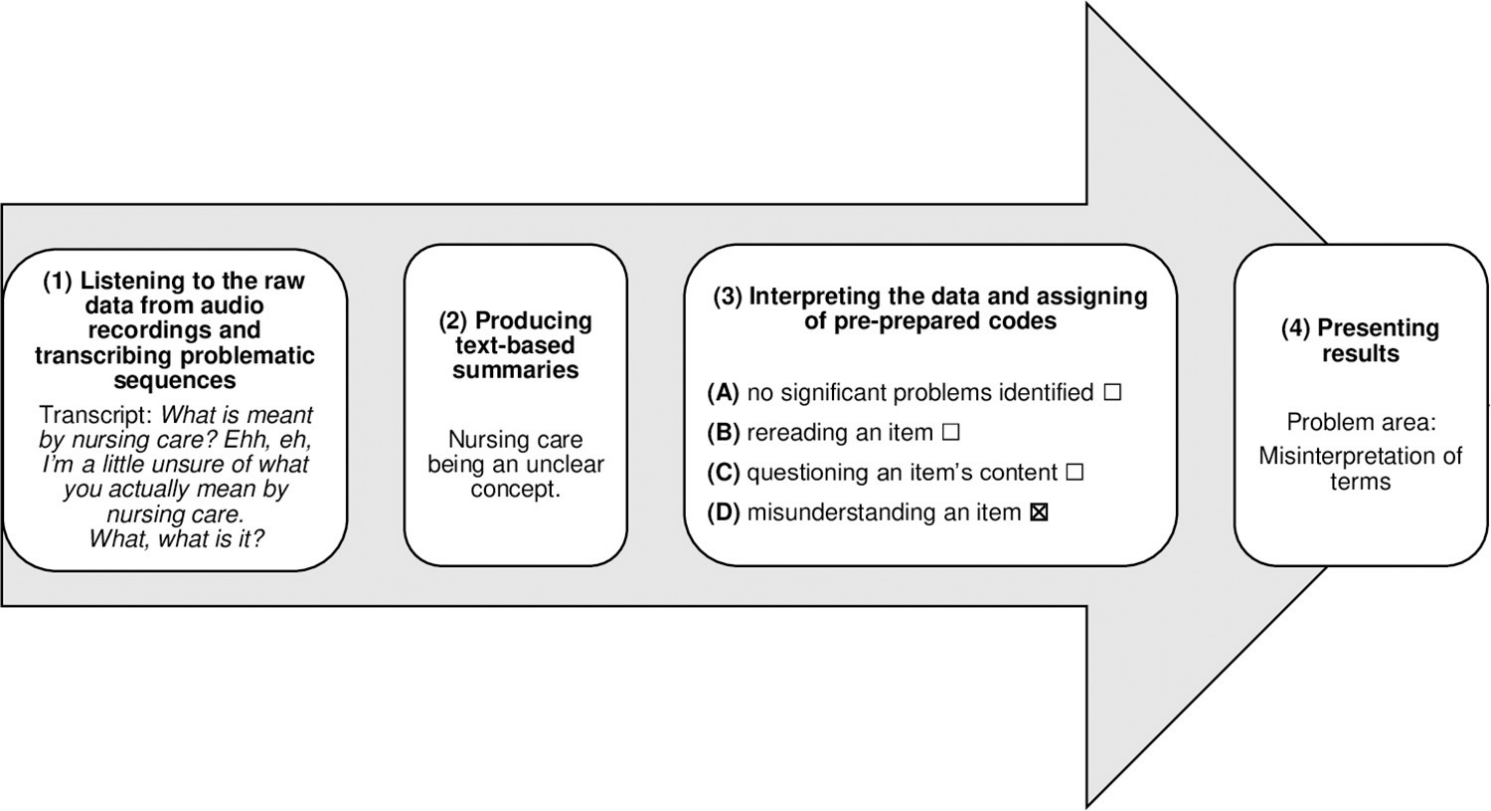
Example of the four steps of analysis of cognitive interviews.

The “inspect-and-repair” model for the analysis of cognitive interviews was used. This model focuses on improving survey questions and on reducing response error in four steps [34]. The four steps of analysis are exemplified in Fig 3.

The first interview was transcribed by an experienced transcriber, and thereafter, the whole research team listened to the first interview while simultaneously reading the transcript. It was agreed that transcribing full interviews did not contribute to the analysis process in regard to identifying problematic areas. Listening carefully and repeatedly was regarded as an adequate analysis method, as tone of voice, pauses, and emphasis all contributed to problem identification. The interviews were analyzed deductively using the Think-aloud protocol with codes inspired by French et al. [35]: (A) no significant problems were identified, (B) rereading an item, (C) questioning an item’s content, and (D) misunderstanding an item. Notes were made after every interview regarding audio quality, main issues, and important comments made by the participant.

All four researchers independently coded and analyzed all ten interviews in the first round. Each researcher transcribed problematic sequences verbatim and took thorough notes that were compared and discussed in the research group until consensus was reached concerning coding and significant problems.

In the second round, the first and second authors analyzed each of the nine interviews independently and thereafter compared their analyses. The other two authors analyzed two randomly selected interviews from each of the two rounds. All four researchers independently analyzed the same four interviews at this last stage of the analysis.

After each round of interviews, intercoder agreement was used to improve the quality of the coding of Think-aloud interviews [36]. In other words, two or more researchers were able to resolve through discussion any coding discrepancies there may have been for the same item and interview.

### Ethical considerations

The study was conducted in accordance with the ethical guidelines laid out in the Helsinki Declaration [37]. According to Swedish law (SFS 2003:460) [38], ethical approval was not required for steps I and II since this part of the study did not involve patients and no sensitive data were elicited. Step III was approved by the Swedish Ethical Review Authority (Reg. no. 2020–05276, and 2021–01982). All participants gave their written informed consent.

## Results

### Step I. Identification of domain and item generation

**Identification of domain.** The definition of “family involvement” used in this study originates from a scoping review of patient and family involvement in adult critical and intensive care settings [12]. The review identified five aspects of family involvement on a continuum from passive to active: (1) family presence, including visits and presence at bedside, (2) family needs being met/family being supported, (3) communication and informational support between family and health care professionals, (4) family participation in decision-making regarding treatment plans and life support, and (5) family contribution to care, such as bathing, massaging, and giving moral support to the patient.

#### Item generation

Two existing questionnaires relevant for the context of inpatient surgical care of the adult person and validated in Swedish were identified during the item generation phase in 2019 and then assessed for content and structure: the Patient Involvement Questionnaire (PIQ) [39] and the Swedish Family Satisfaction Intensive Care Questionnaire (SFS-ICQ) [40]. Permission to reuse items from these two questionnaires were obtained from the originators of the instruments (i.e., from Dr. Judy Arnetz for PIQ, and Mr. Johan Thermaenius and colleagues for SFS-ICQ). Items from these questionnaires were selected based on their correspondence to the domain definition of family involvement outlined above. The item selection phase and the authors’ judgment of item correspondence to the domains are illustrated in Table 2.

**Table 2:** Item selection phase in Step I. Questions selected from the Patient Involvement Questionnaire (PIQ) and Swedish Family Satisfaction Intensive Care Questionnaire (SFS-ICQ) and their correspondence to the domains used in the Family Involvement in Care Questionnaire (FICQ).

	DOMAIN^a^					
	Presence and visitation^b^	Family needs being met^b^	Communi-cation^b^	Participation in decision-making^b^	Contribution to care^b^	Item no^c^
**PIQ** ^**d**^						
What does patient participation mean to you? To what extend do you agree that the following aspects are important:						
That you as a patient receive clear information?			X			1
That you as a patient ask questions?			X			2
That you as a patient, is involved in making decisions about your care and treatment?				X		3
That you, as a patient, are involved in discussions about your care and treatment?				X		4
That you as a patient express your views?				X		5
Did you have the opportunity to ask questions about your diagnosis/conditions?			X			8
Did you understand the information that you received about your diagnosis/conditions?			X			9
Were you involved in discussions which medical examinations/treatments where to be carried out in your case?				X		11
Did you discuss the goal of your treatment with your doctor?				X		12
Were you involved in planning your care after discharge, i.e. what would happen when you were discharged from the hospital?				X		13
Would you have liked to have been more involved in planning your care after discharge?				X		14
Were you treated with respect?		X				16
Were the doctors and healthcare staff responsive to your needs/wishes?		X				17
Have you been able to get in contact with the doctor´s practice/hospital ward when you felt the need to do so?		X				18
Did you wish to have been more involved in your care during your time at the hospital?					X	25
On a scale from 1 to 10, how satisfied are you with your participation in the care of your heart attack/heart problem?					X	26
**SFS-ICQ** ^**e**^						
I received sufficient information from the doctors regarding my family member’s care.			X			10
As my family member could not speak for himself/herself, the staff asked me about my opinion of his/her supposed will.				X		15
I was treated well by the staff in the ICU.		X				19
I had confidence in the nurses that worked in the ICU.		X				20
I received the support that I needed during the time of care in the ICU.		X				21
The ICU staff gave me the opportunity to visit my family member as often as I desired.	X					22
The ICU staff gave me the opportunity to actively participate in the care of my family member to the extent that I desired.					X	23
The information that I received from the doctors was easy to understand.			X			24
**Total number of items in each domain**	1	6	6	8	3	

^a^Domain as defined and published by Boateng et al. [31]

^b^Domains of family involvement based on the definition of and published by Olding et al. [12]

^c^Corresponding numbers to the FICQ numbering as shown in the current study

^d^Original version of items translated from Swedish to English published by Arnetz et al. [39]

^e^Original version of items translated from Swedish to English published by Thermaenius et al. [40]

The PIQ measures patient involvement in patient care following myocardial infarction. The questionnaire consists of 54 items, including demographic questions, divided into six subscales [39]. The scales in the PIQ deemed relevant for this study were “Patient involvement”, “Information”, “Patient needs”, and “Treatment planning”. The scales “Illness experience” and “Activity” were considered to be too patient specific, whereas questions in regard to “Patient needs”, for example, could sometimes be changed to “Family needs”.

The SFS-ICQ is a measurement of family satisfaction with care and is used as a proxy for patient satisfaction with care in the intensive care setting [40]. It has been validated in general and cardiothoracic ICU care in Sweden. It consists of 24 scaled items divided into six areas. Since the validation study of the SFS-ICQ, an additional item regarding asking the family for the patients presumed wishes has been added and is used in clinical practice. The four areas in the SFS-ICQ relevant to this study were “Treatment/Care of the patient”, “Participation in care”, “Reception of family members”, and “Information”. The area “Environment” was deemed to be too ICU specific, and the area “Overall satisfaction” was related only to satisfaction with care and therefore did not correspond well with the definition of family involvement used in this study. Some items in the PIQ and SFS-ICQ were similar, mainly regarding information and how the person was received by health care providers. After obtaining the original authors’ consent, an initial pool of items was selected from the PIQ (16 items) and SFS-ICQ (8 items) and changed in form and wording to fit the context and target population.

One additional item was generated through discussion within the research group, asking for the rating of the importance of participation in nursing care. The added item followed the structure of the items in the PIQ and was intended to cover the domain “contribution to care”. The first version of the questionnaire consisted of 25 items. Each item was phrased, as in the SFS-ICQ, as a statement with response options on a four-point Likert scale: (1) “I fully agree”, (2) “I largely agree”, (3) “I somewhat agree”, and (4) “I fully disagree”. There was also a “Not relevant” option. One open-ended question was included, giving the respondent an opportunity to share additional views on what they considered important regarding family involvement. In addition, demographic data were collected regarding age, gender, and the family member’s relationship to the patient. All versions of the questionnaire tested in steps II and III are shown in Table 3.

**Table 3:** The three tested versions of Family Involvement in Care Questionnaire: Problems identified and alterations made.

Item	Items in step II *Expert groups rating of relevance and understandability* (Instrument of origin)	I-CVI	Problem identified in step II	Altera-tions made by the research team	Items in step III, round 1 *First round of cognitive interviews*	Problem identified in step III, round 1^a^ (n)	Altera-tions made by the research team	Items in step III, round 2 *Second round of cognitive interviews*	Problem identified in step III, round 2^b^ (n)	Altera-tions made by the research team	Final items
	What does involvement in care mean to you as a family member?		Term *family* questioned	The question rephrased into a statement	I am involved in the care of my family member when:		The statement rephrased	I feel involved in the care of my family member when I get:		Items excluded	
1	Receiving clear information (PIQ)	1.00		Item 1–6 reworded, a new item (item 7) added	I receive clear information	3	The sequence of items reordered, items reworded	clear information	4		
2	Being able to ask questions (PIQ)	1.00	Understand-ability questioned		I get the opportunity to ask questions	3		the opportunity to ask questions	4		
3	Participating in decision-making about my family member’s care and treatment (PIQ)	0.86			I get to participate in decision-making about my family member’s care and treatment	4		to participate in decision-making about my family member’s care and treatment	5		
4	Participating in discussions about my family member’s care (PIQ)	0.86			I get to participate in discussions about my family member’s care	4		to participate in discussions about my family member’s care	5		
5	Having the opportunity to express my views (PIQ)	1.00			I have the opportunity to express my views	4		the opportunity to express my views	4		
6	Participating in the nursing care of my family member	1.00	Term *nursing care* questioned		I get to participate in the nursing care of my family member	4		to participate in the nursing care of my family member	5		
7					My own needs are attended to by the staff	3		my own needs attended to by the staff	5		
8	I had the opportunity to ask questions about my family member’s illness/condition (PIQ)	1.00		None	*	0	None	*	0	None	I had the opportunity to ask questions about my family member’s illness/condition
9	I understood the information I received regarding my family member’s illness/condition (PIQ)	1.00		None	*	0	None	*	0	None	I understood the information I received regarding my family member’s illness/condition
10	I received sufficient information from the staff at the hospital regarding my family member’s care (SFS-ICQ)	1.00	Term *care* and *staff at the hospital* questioned	Item reworded	I received sufficient information regarding my family member’s care	1	None	*	0	None	I received sufficient information regarding my family member’s care
11	I participated in the discussion about which examinations/treatments should be done (PIQ)	0.86	Understand-ability questioned by one expert	None	*	1	None	*	0	None	I participated in the discussion about which examinations/treat-ments should be done
12	I participated in the discussion about the goal of my family member’s treatment (PIQ)	0.86	Suggested to be analogous to item 10 by one expert	None	*	1	None	*	1	None	I participated in the discussion about the goal of my family member’s treatment
13	I participated in the planning of my family member’s aftercare, that is, what would happen when my family member was discharged from the hospital (PIQ)	1.00		None	*	1	None	*	1	None	I participated in the planning of my family member’s aftercare, that is, what would happen when my family member was discharged from the hospital
14	I would have liked to be more involved in planning my family member’s aftercare (PIQ)	1.00		None	*	0	None	*	1	None	I would have liked to be more involved in planning my family member’s aftercare
15	When my family member was unable to express their wishes, the staff asked me for my opinion on their presumed will (SFS-ICQ)	1.00	Term *staff* questioned	None	*	3	None	*	3	Exempli-fying situations	When my family member was unable to express their wishes, for example when sedated in the ICU, the staff asked me for my opinion on their presumed will
16	The staff treated me with respect (PIQ)	1.00		None	*	0	None	*	0	None	The staff treated me with respect
17	The staff at the hospital were responsive to my needs/wishes (PIQ)	1.00	Term *staff at the hospital* questioned	Items reworded	The staff were responsive to my needs/wishes	2	None	*	0	None	The staff were responsive to my needs/wishes
18	It was easy to get in touch with the staff at the hospital when I felt the need (PIQ)	1.00			It was easy to get in touch with the staff when I felt the need	0	None	*	0	None	It was easy to get in touch with the staff when I felt the need
19	I was well received by the staff at the hospital (SFS-ICQ)	1.00			I was well received by the staff	0	None	*	0	None	I was well received by the staff
20	I felt confident in the staff at the hospital (SFS-ICQ)	0.86			I felt confident in the staff	1	None	**	0	None	I felt confident in the staff
21	I received the support I needed during the time at the hospital (SFS-ICQ)	1.00	Term *support* questioned		I received the support I needed during my family member’s care period	0	None	*	2	Specifying type of support	I received the emotional support I needed during my family member’s care period
22	I was given the opportunity to be with my family member as often as I wanted (SFS-ICQ)	1.00		None	*	0	None	*	0	None	I was given the opportunity to be with my family member as often as I wanted
23	I was given the opportunity to participate in the nursing care of my family member to the extent I wanted (SFS-ICQ)	1.00	Term *nursing care* questioned	None	*	3	None	*	2	Rewording item, changing *nursing care* into examples	I was given the opportunity to help my family member with everyday chores he/she usually manages on his/her own (e.g. going to the toilet, shaving/brushing hair or helping with meals)
24	The information I received from the staff at the hospital was easy to understand (SFS-ICQ)	0.86	Term *staff at the hospital* questioned, item sugge-sted to be analogous to item 8	Items excluded							
25	I wish I could have been more involved in my family member’s care while in the hospital (PIQ)	0.71	Regarded as similar to item 26								
26	I am satisfied with my involvement in the care of my family member (PIQ)	0.86	Term *satisfaction* questioned								
Open-ended questions:		If you would like to leave a comment on any of your answers in the questionnaire, please do so here							**		
		If you have any further point of views on involvement that you consider important, please share these here							**		

^a^Total number of participants = 10, giving a maximum score of 20 problems for each item

^b^Total no of participants = 9, giving a maximum score of 18 problems for each item

*Same wording as in previous step

**Same wording throughout all steps

I-CVI = Item Content Validity Index; ICU = Intensive Care Unit; PIQ = Patient involvement questionnaire; SFS-ICQ = Swedish Family Satisfaction Intensive Care Questionnaire

After the evaluation by experts in step II, the authors decided that the aspect of “having needs met and being supported” was not represented among the initial items where the participant was asked to rate the importance of the domains. An item rating the importance of having one’s own needs met was therefore added. An additional open-ended question was also added at this stage, giving respondents an opportunity to elaborate on any specific answers they had given to the prior items. The second version of the questionnaire, tested in the cognitive interviews in step III, consisted of 23 scaled items and two open-ended questions. The questionnaire instructions indicated, “Please note that the questions only refer to the time when your family member was taken care of in the cardiothoracic surgical department”. Instructions for the first seven items indicated, “these questions concern aspects important for your sense of being involved in your family members’ care”. Instructions for the following 16 items indicated, “these questions concern how you were involved in your family members’ care”.

### Step II. Content validity

#### Evaluation by experts

The I-CVI varied between 0.71–1.00, and the value for S-CVI/Averaging was 0.90. The I-CVI of each individual item is shown in Table 3. One item was excluded due to its low I-CVI (I-CVI = 0.71). The free-text comments on relevance were discussed by the research group, resulting in the rewording of twelve items and the exclusion of two. Since comprehensibility should be evaluated by the target group, the experts’ comments regarding item understandability were discussed within the research group but did not lead to the exclusion of items at this point.

### Step III. Pretesting questions

#### Cognitive interviews with Think-aloud: Round one

When conducting the analysis of the interviews, three main problem areas with the questionnaire were identified as misinterpretation of the purpose of the questions related to important aspects of family involvement, misinterpretation of the term “nursing care,” and underuse of the “Not relevant” answer option. A problem regarding the probing strategy was also identified. Rereading an item was not identified as a problem. Therefore, the participants could potentially have had two problems with one item, i.e., misunderstanding an item and/or questioning its content, making the maximum number of problems in round one 460. No participant had more than one problem per item, giving a total number of 38 problems in round one.

#### Defining family involvement

Regarding Items 1–7, the instructions asked the participants about what was important for them to be involved in their family members’ care in general terms. However, as these seven items was frequently misinterpreted or questioned, they were considered to be the main problem area after the first round. Problems were mainly misinterpretations. Participants described the circumstances during the specific care period instead of rating how important these aspects were for the participant’s sense of being involved.

Well … involved in care… Received clear information… Um, I hardly got that, any information. I’d probably say that I somewhat agree. (Participant 1, round one, answering Item 1)

Questioning of item content in round one concerned the relevance of participating in decision-making and the items being closely linked to one another.

I actually think those questions overlap a bit. Umm, but, umm well… Umm, I suppose it’s good. Of course, you feel involved when you are part of the discussion. Umm. I largely agree with that. (Participant 2, round one, answering Item 4)

The extent of the problems with Items 1–7, interpreted as being related to the questionnaire instructions in this first round, indicated that alterations were needed. This was confirmed by the answers to the final open-ended questions and in the retrospective probe of problematic items. These items were reworded, at the end of the version used in round two, and instructions for these items were clarified with the statement “the questions on this page concern what you as a family member regard as important for you to feel involved in the care of your family member”.

#### Misinterpretation of the term “nursing care”

The item asking whether the family member had been given the opportunity to participate in nursing care to the extent he or she wished was questioned by one participant: “Didn’t you ask this before?” (Participant 3, round one, answering Item 23). The participant probably referred to the defining item (Item 6 in Table 3) “I am involved in the care of my family member when I get to participate in the nursing care of my family member”. Item 23 was also misinterpreted by two other participants, where one of them disregarded the aspect of “to the extent I wished”. The other case of the term participation in nursing care not being understood in the way it was intended to be was when the participant referred to participation in nursing care at home and not in the hospital.

Well, I must say, nursing care, that was when she got home so, I fully agree, because it was necessary for me to participate in nursing care because complications meant that she needed my help. I fully agree to that. (Participant 4, round one, answering Item 23)

#### Underuse of the “Not relevant” option

On occasions, participants said that an item was irrelevant to their situation but chose the answer “I somewhat agree” or “I fully disagree” instead of choosing the option “Not relevant,” with the result that a possibly neutral experience appeared negative.

What should I answer to this one [this question]? That was truly hard. Umm … No, I fully disagree, it must be that. That one was slightly funny because it might not represent how it was at all. However, it could be others who, other situations, ohh. No, I can’t answer that, it’s not right. (Participant 5, round one, answering Item 15)

The response options for all items in the questionnaire were shortened to (1) “Fully agree”, (2) “Largely agree”, (3) “Somewhat agree”, and (4) “Fully disagree”, possibly eliminating the risk of misinterpreting answer options. The option “Not relevant” was not altered.

Regarding involvement in care, the participants identified a lack of questions related to their receiving information prior to elective surgery and information on whom to turn to after the care period at the hospital. The research group thought that these aspects were covered by items inquiring whether they had received sufficient information leading to no alterations at this point.

All items with problems in round one were discussed by the research group. Given the extent of the changes to the first seven items, changes to items related to nursing care were set aside to see whether these problems persisted in the second round.

### Cognitive interviews with Think-aloud: Round two

In the second round, the problems identified in round one were confirmed. Rereading an item was not identified as a problem in this round, making the potential maximum number of problems in round two 414. The most prominent problem concerned Items 1–7, where the participant was asked to define important aspects of involvement in general terms and not to relate their answers to the specific care period. The total number of problems identified in round two was 42, whereas 32 concerned Items 1–7.

#### Defining family involvement

Problems with misinterpreting the purpose of Items 1–7 remained after the second round of cognitive interviews, and they were therefore excluded from the questionnaire.

Yes, but there you are, I think we were just grateful for the care given, so I don’t think, I don’t think we questioned or participated in discussions in that way. Because, you know, now this is one of those questions you can interpret a little from my point of view, “participate in discussions”, that is … If I’d wanted to, I would probably have gotten the opportunity. (Participant 6, round two, answering Item 4)

#### Misinterpretation of terms

Consistent with the results in round one, the term “nursing care” was considered problematic, a problem that was also pointed out by the expert group in step II.

No, that’s not true at all, I … That’s taken care of by the professionals at the hospital, so I don’t need to get involved with that. Fully disagree. (Participant 7, round two, answering Item 23)

In the final version of the questionnaire, the term “nursing care” was replaced with examples of basic care activities. The term “support” was also questioned, as it was ambiguous.

Well, thinking of this in terms of a questionnaire construct … It’s actually an open question, a very open question. What does it mean? What kind of support could one get? The question is kind of unclear then, in my opinion. “The support I needed” … A hug?–No, I did not get that. *Laughs* You understand? It’s sort of unclear, what do you need? Umm, but in this case I suppose I got what I needed from the staff, kind of, so I suppose I would have answered largely agree or maybe … yes, I would have. (Participant 8, round two, answering Item 21)

In response to this observation, it was specified that the “support” referred to was emotional support.

#### Underuse of the “Not relevant” option

As in round one, when faced with items that the participants regarded as not relevant for them, they chose the option “Fully disagree,” making it a negative experience instead of a neutral one.

No, well he could speak for himself. *Rereads the question. * No that * rereads the question again*, well I don’t know what to answer, but he could speak for himself. Fully disagree then. Must be right, right? Yes, that one is it. (Participant 9, round two, answering Item 15)

As is clear from the response above, the item regarding whether the family member had been given an opportunity to express the patient’s presumed will was problematic. Therefore, an example of when patients might be unable to express their will (e.g., while sedated in the ICU) was added, and the “Not relevant” option was explained in the instruction to the questionnaire and changed to bold font.

#### Retrospective probes- possibly identifying unverbalized problems

Answers to the retrospective probes in round two revealed that three of the nine participants had difficulty limiting their reported experiences to the specific care period in the cardiothoracic surgical department. “Discussions” and “decision-making” were reported as somewhat confusing concepts in retrospect. One person reported missing items of information regarding possible outcomes and persisting issues for the patient at home.

What I think is that I should have been given the opportunity to express my point of view on my experiences in the time afterward. Because I don’t know, I usually say to my wife, you are not the same as you were before you went away. That is, her temper changed and umm, these things. There were no questions about that. (Participant 10, round two, answering probe of problematic/missing items)

Potential missing items proposed by participants were discussed by the research group but deemed to be covered by items relating to information. The final version of the questionnaire consisted of 18 items: 16 items with a four-point Likert scale and two open-ended items. An overview of the problems and alterations made in the three tested versions of the Family Involvement in Care Questionnaire (FICQ) is shown in Table 3. The final version of the FICQ is displayed in the Supporting Information file S1 Appendix.

## Discussion

FICQ was developed as a measure of family involvement in inpatient care. The questionnaire consists of 18 items of which 16 has been reformulated and adapted from two existing instruments, the PIQ [39] and the SFS-ICQ) [40]. FICQ is designed for family members of patients who have received inpatient care, and its content validity has been evaluated in the context of open-heart surgical care. The expert group’s rating of relevance indicated excellent content validity prior to the target group interviews. If the investigation had stopped at that point, the problems for the target population would not have been identified [41]. This indicates that evaluation using the target population is important, and theory- and expert-based instruments are not sufficient.

In this study, having items with two separate purposes was problematic. In the initial questionnaire, important aspects of family involvement were rated in a general manner (Items 1–7 in Table 3). These items were followed by items evaluating the participant’s rating of being involved during a specific care period. This made some participants uncertain about the procedure. It was therefore decided not to mix questions with different purposes, and Items 1–7 were excluded. The original version of these items has not been reported as problematic [39].

Active family involvement in hospital care can contribute to a reduction in surgical complications, early detection of delirium, and facilitation of early mobilization [7] and should therefore be encouraged. Personal hygiene [10], oral care, and mobilization [7, 10] are examples of basic care activities the family can be offered to contribute to in a hospital setting. However, the term “nursing care” was problematic for some participants in this study, as was also found by Thermeanius et al. [40]. Nursing care was seen by some family members as a professional domain in the hospital [40], or as only becoming the responsibility of the family when the patient was discharged [42]. Perhaps the term “basic care”, as described in previous litterature [7, 10], is better suited for this purpose. The family members can be given concrete examples of caring tasks that they can carry out in the hospital environment. In this study, the term “nursing care” was ultimately changed to examples of caring activities, e.g., helping with meals (see Item 23 in Table 3).

Another problematic term in our questionnaire was the “support” given by the staff to the family. Patients have described recovery after surgery as possibly harder on the family than on the patients themselves [18]. Previous research has explored the importance of supporting the whole family when one family member has undergone surgery [42]. In our study, some participants questioned the relevance of evaluating the support they themselves had received or asking what kind of support they could have had. It has previously been shown that family members can find it difficult to identify their own needs during a loved one’s hospitalization, except for the need for information [1]. The types of support nurses provide to family members have been categorized as either cognitive or emotional [43]. Cognitive support entails education and information to support coping, while emotional support has been described as helping the family member handle the range of emotions experienced when a family member is ill [43]. Informational support has been described as the most important aspect of supporting the family [44] and is adequately covered in the FICQ. Therefore, the item regarding support (Item 21 in Table 3) was modified to specify “emotional support”.

Alterations were made after the second round of cognitive interviews (see Items 15, 21 and 23 in Table 3), and Items 1–7 were excluded. The remaining items had a total of 10 problems, with one item having three problems. One could therefore argue that a third round of cognitive interviews would have been required to evaluate these changes [45]. However, as Willis [34] states, when conducting cognitive interviews, investigators must at some point decide that enough is enough. It was thus decided that these items could be tested further in a future quantitative psychometric study with a focus on scale evaluation [31].

### Strengths and limitations

The initial three steps in best practices guidelines for questionnaire development were followed [31]. Attempts were also made to achieve the goals of trustworthiness and credibility, transferability, dependability, and confirmability as described by Shenton [45]. Credibility was strengthened by using well-established research methods. All authors are nurses with experience in intra- and/or postoperative care settings. The first and last authors are very familiar with the specific context where the study took place as they were employees at the clinic, but they had no ongoing or previous interactions with neither the family members being interviewed nor the expert nurses that evaluated the questionnaire. Sampling for the cognitive interviews was random, which is a strength in some instances [45]. Triangulated qualitative methods were used to test validity through the convergence of information from health care providers and family members. One person conducted the interviews, thus contributing to the standardization of the interviews. All four authors analyzed interviews from both rounds using a structured protocol [35], strengthening credibility both in terms of frequent debriefing and the trustworthiness of the analysis. The six aspects enabling the assessment of transferability have been accounted for [45]. Regarding dependability and confirmability, our aim has been to preserve an audit trail that will be helpful as a framework for future investigations relating to cognitive interviews on videoconferencing platforms. We found the method of using cognitive interviews in the validation process to be very useful in informing further questionnaire development.

Our study does have some limitations. One was that the researcher who conducted the interviews was a doctoral student with limited experience from interviewing prior to this study. She was provided continuous support regarding the interview technique from the second author which have prior experience in using the Think-aloud method, as well as supervision from the other two authors. One was the limited ability to observe and assess nonverbal communication when using online videoconferencing platforms rather than face-to-face interviews [46]. Not all participants chose to have their video on. The practice of allowing long pauses was compromised, as pauses could indicate technical problems. In the first round, the intention was to conduct Think-aloud interviews using no verbal probing besides the prompt “think out loud”. Following this strategy consistently was difficult in the first round, as silence could be an indication of a cognitive problem of the participant or technical problems such as losing internet connection. With the broader probing strategy in the second round of cognitive interviews, a few new problems were identified, such as the fact that three out of the nine participants in the second round had difficulties limiting their responses to the one care period asked about. Several previously identified problems were confirmed, such as defining family involvement and misinterpreting the term nursing care. This suggests that the method is suitable and that a structured concurrent and retrospective probing strategy could be beneficial when using videoconferencing platforms such as Zoom. There were, in fact, some instances of technical problems with interrupted connections and audio failure. However, previous qualitative research on the use of Zoom indicates that technical problems can contribute to building rapport between the investigator and the study participant [47]. Such problems can generate collaboration, which contributes to building trust, a stable ground when collecting qualitative data [47].

The technical demands on participants led to one person declining to participate, indicating an unequal aspect of technical requirements on participants. The aspect of availability to presumptive study participants is addressed by Carter et al. [48], that states availability does not decrease but rather changes when interviews are conducted online. The solution to the technical demands on participants could be to provide all necessary equipment and travel to the participants’ homes to set up the technology [49]. This does nevertheless have some drawbacks that affect the advantages of cost, time, and efficiency [47].

One limitation of this study is that a potential generic questionnaire measuring family involvement in inpatient care was tested in only one inpatient care setting. Therefore, the questionnaire needs further psychometric testing in various settings before any conclusions regarding validity and reliability as a generic inpatient measure can be drawn. However, as previously stated, the inpatient cardiothoracic surgical care pathway is diverse, including different care settings in one, and was therefore chosen in this first step of development and validation. The items in FICQ could be perceived as only relevant to critical care, such as after myocardial infarction or while in intensive care. This was, however, not found to be problematic in our study by either experts or target group participants. Questions developed for patient participation in a cardiology context [39] adapted for family involvement were regarded as valid by both experts and the targeted population, with the exception of items 1–7 which therefore were excluded.

## Conclusions

The usage of this cognitive validation model resulted in a refined questionnaire, ready to be tested quantitatively regarding reliability and further aspects of validity. Direct target population feedback was effective to receive prior to administration to a large sample of family members. Before using FICQ in a clinical setting, further research needs to be conducted to determine whether the questionnaire is psychometrically sound. After being validated and reliability tested, the FICQ can be used to provide clinicians and policymakers with information on how families are supported, informed, and treated in connection with inpatient care. The extent of their involvement could be used as an indicator of the quality of care and used when evaluating interventions that aim to increase family involvement in care.

## Supporting information

S1 Appendix Final version of the Family Involvement in Care Questionnaire (FICQ). (PDF)
