# Supplementary material for: The Family Involvement in Care Questionnaire—An instrument measuring family involvement in inpatient care
Source: PLoS One. 2023 Aug 15;18(8):e0285562. doi: 10.1371/journal.pone.0285562 (PMC10426968; doi:10.1371/journal.pone.0285562)
Supplement: S1 Appendix — (PDF) [file pone.0285562.s001.pdf]

Family Involvement in Care Questionnaire (FICQ)

These questions is about how you as a family member has been involved in the care of your family member when he or she was amitted at the NNNNNNN clinic

Answer each statement by ticking the most relevant box. If you feel unsure, tick the box that seems to be most appropriate. If the statement is irrelevant please tick the "Not relevant" box. Only tick one box per statement.

	Fully agee	Largely agree	Somewhat agree	Fully disagree	Not relevant
1. I had the opportunity to ask questions about my family member's illness/condition	☐	☐	☐	☐	☐
2. I understood the information I received regarding my family member's illness/condition	☐	☐	☐	☐	☐
3. I received sufficient information regarding my family member's care	☐	☐	☐	☐	☐
4. I participated in the discussion about which examinations/treatments should be done	☐	☐	☐	☐	☐
5. I participated in the discussion about the goal of my family member's treatment	☐	☐	☐	☐	☐
6. I participated in the planning of my family member's aftercare, that is, what would happen when my family member was discharged from the hospital	☐	☐	☐	☐	☐
7. I would have liked to be more involved in planning my family member's aftercare	☐	☐	☐	☐	☐

©FICQ version 2022-11-25
Ongoing validation process, not for use
Sundqvist A-S, Drakenberg A, Ericsson E, Prignitz-Sluys K
Contact: ann-sofie.sundqvist@oru.se

Family Involvement in Care Questionnaire (FICQ)

	Fully agee	Largely agree	Somewhat agree	Fully disagree	Not relevant
8. When my family member was unable to express their wishes, for example when sedated in the ICU, the staff asked me for my opinion on their presumed will	☐	☐	☐	☐	☐
9. The staff treated me with respect	☐	☐	☐	☐	☐
10. The staff were responsive to my needs/wishes	☐	☐	☐	☐	☐
11. It was easy to get in touch with the staff when I felt the need	☐	☐	☐	☐	☐
12. I was well received by the staff	☐	☐	☐	☐	☐
13. I felt confident in the staff	☐	☐	☐	☐	☐
14. I received the emotional support I needed during my family member's care period	☐	☐	☐	☐	☐
15. I was given the opportunity to be with my family member as often as I wanted	☐	☐	☐	☐	☐
16. I was given the opportunity to help my family member with everyday chores he/she usually manages on his/her own (e.g. going to the toilet, shaving/brushing hair or helping with meals)	☐	☐	☐	☐	☐

©FICQ version 2022-11-25
Ongoing validation process, not for use
Sundqvist A-S, Drakenberg A, Ericsson E, Prignitz-Sluys K
Contact: ann-sofie.sundqvist@oru.se

17. If you would like to leave a comment on any of your answers in the questionnaire, please do so here.

……
……
……
……
……
……
……
……
……
……
……
……

18. If you have any further point of views on involvement that you consider important, please share these here

……
……
……
……
……
……
……
……
……
……
……
……

©FICQ version 2022-11-25
Ongoing validation process, not for use
Sundqvist A-S, Drakenberg A, Ericsson E, Prignitz-Sluys K
Contact: ann-sofie.sundqvist@oru.se
